# Physical Activity, Mediterranean Diet and Biomarkers-Assessed Risk of Alzheimer’s: A Multi-Modality Brain Imaging Study

**DOI:** 10.4236/ami.2014.44006

**Published:** 2014-10

**Authors:** Dawn C. Matthews, Michelle Davies, John Murray, Schantel Williams, Wai H. Tsui, Yi Li, Randolph D. Andrews, Ana Lukic, Pauline McHugh, Shankar Vallabhajosula, Mony J. de Leon, Lisa Mosconi

**Affiliations:** 1 ADM Diagnostics, Chicago, USA; 2 Department of Psychiatry, New York University School of Medicine, New York, USA; 3 Citigroup Biomedical Imaging Center, Weill Cornell Medical College, New York, USA

**Keywords:** Alzheimer’s Disease, Mediterranean Diet, Physical activity, PET Imaging, Amyloid, Glucose Metabolism, MRI, Early Detection, Brain Aging

## Abstract

Increased physical activity and higher adherence to a Mediterranean-type diet (MeDi) have been independently associated with reduced risk of Alzheimer’s disease (AD). Their association has not been investigated with the use of biomarkers. This study examines whether, among cognitively normal (NL) individuals, those who are less physically active and show lower MeDi adherence have brain biomarker abnormalities consistent with AD.

**Methods:**

Forty-five NL individuals (age 54 ± 11, 71% women) with complete leisure time physical activity (LTA), dietary information, and cross-sectional 3D T1-weigthed MRI, ^11^C-Pittsburgh Compound B (PiB) and ^18^F-fluorodeoxyglucose (FDG) Positron Emission Tomography (PET) scans were examined. Voxel-wise multivariate partial least square (PLS) regression was used to examine the effects of LTA, MeDi and their interaction on brain biomarkers. Age, gender, ethnicity, education, caloric intake, BMI, family history of AD, Apolipoprotein E (APOE) genotype, presence of hypertension and insulin resistance were examined as confounds. Subjects were dichotomized into more and less physically active (LTA+ vs. LTA−; n = 21 vs. 24), and into higher vs. lower MeDi adherence groups (n = 18 vs. 27) using published scoring methods. Spatial patterns of brain biomarkers that represented the optimal association between the images and the groups were generated for all modalities using voxel-wise multivariate Partial Least Squares (PLS) regression.

**Results:**

Groups were comparable for clinical and neuropsychological measures. Independent effects of LTA and MeDi factors were observed in AD-vulnerable brain regions for all modalities (p < 0.001). Increased AD-burden (in particular higher A*β* load and lower glucose metabolism) were observed in LTA− compared to LTA+ subjects, and in MeDi− as compared to MeDi+ subjects. A gradient effect was observed for all modalities so that LTA−/MeDi− subjects had the highest and LTA+/MeDi+ subjects had the lowest AD-burden (p < 0.001), although the LTA × MeDi interaction was significant only for FDG measures (p < 0.03). Adjusting for covariates did not attenuate these relationships.

**Conclusion:**

Lower physical activity and MeDi adherence were associated with increased brain AD-burden among NL individuals, indicating that lifestyle factors may modulate AD risk. Studies with larger samples and longitudinal evaluations are needed to determine the predictive power of the observed associations

## 1. Introduction

Alzheimer’s disease (AD) is the most common cause of dementia and a major public health problem. Given the current lack of disease-modifying treatments, and the increasing awareness that symptoms develop over many years, there has been growing interest in identifying effective strategies for prevention or delay of onset [1]. While a small percentage of early-onset AD cases are caused by genetic mutations, the most common late-onset AD is a multi-factorial disease most likely caused by the complex interplay of genetic risk factors and environmental factors [2]. There is growing evidence to suggest that diet and physical activity, two major targetable lifestyle factors, may play a role in modulating risk of AD. However, the biological mechanisms underlying the relationship between lifestyle, brain aging and AD are largely unexplored.

Pathologically, AD is characterized by the gradual accumulation of amyloid-beta (A*β*) plaques, neurofibrillary tangles and neuronal loss in selectively vulnerable brain regions, which takes place over decades [3]. These changes can be visualized *in vivo* by means of brain imaging [3]. Imaging-based AD biomarkers offer a unique opportunity to understand how lifestyle can promote healthy brain aging prior to symptoms onset.

Epidemiological and clinical studies have provided evidence that higher adherence to a Mediterranean diet (MeDi)-type pattern is associated with reduced risk of AD [4]-[8]. Magnetic Resonance Imaging (MRI) studies have shown that lower adherence to the MeDi is associated with reduced cortical thickness (*i.e.*, increased atrophy) of key brain regions for AD [9], and increased cerebrovascular disease burden in the elderly [10] [11].

Physical activity has also been associated with increased physical and mental health throughout life [12] [13]. A similar perspective has emerged in AD research, as epidemiological studies have shown a relationship between physical activity and reduced risk of AD [14], which has been corroborated by biomarker evidence for larger brain volumes and lower A*β* burden in physically active compared to sedentary subjects [15]-[18].

Clinical studies that examined the combination of diet and physical activity showed that these lifestyle factors were independently associated with reduced risk for AD, although their combination did not result in further risk reductions relative to each factor alone [14]. To our knowledge, there are no previous studies that examined the combination of diet and physical activity on *in vivo* biomarkers of disease, particularly using a multi-modality approach, or with consideration of the recently re-conceptualized preclinical stages of AD [2]. The present brain imaging study examines A*β* load on ^11^C-Pittsburgh Compound-B (PiB) Positron Emission Tomography (PET), glucose metabolism (CMRglc, a proxy of neuronal activity) on ^18^F-fluorodeoxyglucose (FDG) PET, and gray matter volumes on MRI (*i.e.*, a proxy of neuronal loss) to test the effects of physical activity, MeDi and their combination among cognitively normal adults.

## 2. Methods

### 2.1. Participants

Among a larger pool of clinically and cognitively normal (NL) individuals participating in longitudinal brain imaging studies at New York University (NYU) Langone School of Medicine, this study was based on a sub-set of 62 NL participants who participated in a lifestyle survey between 2013-2014. Subjects were derived from multiple community sources, including individuals interested in research participation, family members and caregivers of impaired patients. All subjects provided written informed consent to participate in this IRB approved study. Individuals with medical conditions or history of conditions that may affect brain structure or function, *i.e.* stroke, diabetes, head trauma, any neurodegenerative diseases, depression, hydrocephalus, intracranial mass, and infarcts on MRI, and those taking psychoactive medications were excluded. Subjects were 25 - 71 year old, with education ≥ 12 y, Clinical Dementia Rating (CDR) = 0, Global Deterioration Scale (GDS) ≤ 2, Mini Mental State Examination (MMSE) ≥ 28, Hamilton depression scale < 16, Modified Hachinski Ischemia Scale < 4 and normal cognitive test performance for age and education [19]. Study analyses focused on 45 participants who fulfilled our inclusion criteria and completed all clinical, MRI, PiB-and FDG-PET exams, physical activity and dietary questionnaires within 6 months of each other. While all subjects were normoglycemic young adults, insulin sensitivity was calculated using the Homeostasis Model Assessment (*HOMA*) [20]. Presence of hypertension was determined based on current antihypertensive treatment or blood pressure assessments (systolic blood pressure ≥ 140 mmHg or diastolic blood pressure ≥ 90 mmHg) [21]. A family history of LOAD that included at least one 1^st^ degree relative whose AD onset was after age 60 was elicited using standardized questionnaires [22]. Apolipoprotein E (APOE) genotypes were determined using standard qPCR procedures [23].

### 2.2. Physical Activity

Physical activity was estimated by means of the Minnesota leisure time activity (LTA) questionnaire [24]. The questionnaire consists of a list of 62 leisure-time physical activities separated into three sections, including walking and miscellaneous (e.g., walking for pleasure, using stairs when an elevator is available, etc.), conditioning exercise (e.g., running, health club activities, etc.), and other exercise (e.g., playing golf, shoveling snow, etc.). Participants were asked if they participated in any of the given activities. For each activity, information was collected on the frequency (how often per month and how many months per year) and duration of engagement (time spent per session). Frequency and duration information were multiplied with an activity-specific intensity code indicating calorie expenditure [24]. The activity-dependent scores were summed to obtain the overall intensity of physical activity per person during the last 12 months and converted to metabolic equivalents (MET), which are independent of body weight [24]. A cut-off of 7.5 MET hours/week (*i.e.*, the energy cost of engaging in 30 min of moderate activity 5 days per week) was used to divide subjects into more (LTA+) and less physically active (LTA−) groups, according to American Heart Association guidelines [25] [26].

### 2.3. Dietary Assessments

Dietary data regarding average food consumption over the prior year were obtained using the 153-item version of Harvard/Willett’s semi-quantitative food frequency questionnaire (SFFQ) [27]. The SFFQ has been used and validated for the determination of nutrient intake in the elderly as well as in young adults, yielding high reliability [27]. Food items were categorized into 30 food groups based on similarities in food and nutrient composition, and intake (g/day) of each food group was calculated by summing the intakes of member food items. The MeDi is characterized by high intake of plant foods; moderate consumption of dairy products, fish, poultry; olive or vegetable oil as the primary dressing; low to moderate intake of wine; low intake of red meat; very low intake of processed foods [7]. Published methods were followed for the construction of MeDi scores [4]-[6] [9]. Briefly, we first regressed caloric intake (in kilocalories) and calculated the derived residuals of daily gram intake for each of the following 7 categories: dairy, meat, fruits, vegetables, legumes, cereals, and fish. Individuals were assigned a value of 1 for each beneficial component (fruits, vegetables, legumes, cereals, and fish) whose consumption was at or above the sex-specific median; a value of 1 for each detrimental component (meat and dairy products) whose consumption was below the median; a value of 1 for a ratio of monounsaturated fats to saturated fats above the median; and a value of 1 for mild to moderate alcohol consumption (1 - 2 drinks per day) [4]-[6] [9]. The MeDi score was the sum of the scores in the food categories, with a greater score indicating greater similarity to the MeDi pattern. Participants were dichotomized into higher and lower MeDi adherence groups [9].

### 2.4. Brain Imaging

All subjects received MRI, PiB- and FDG-PET scans following standardized protocols [28] [29]. The diagnostic MRI study was performed using contiguous 3 mm axial T2-weighted images. These scans were used to rule out MRI evidence of hydrocephalus, intracranial mass, strokes and subcortical gray matter lacunes. The research MRI scan was a volumetric 124 slice T1-weighted Fast-Gradient-Echo acquired in a sagittal orientation as 1.2 mm thick sections. For PET, subjects were positioned in the scanner 60 min after injection of 15 mCi of ^11^C-PiB, and scanned for 30 min in 3D-mode on an LS Discovery or BioGraph PET/CT scanner. The FDG scan was performed 30 min after completion of the PiB scan or on a separate day. After an overnight fast, subjects were injected with 5 mCi of ^18^F-FDG, positioned in the scanner 35 min after injection, and scanned for 20 min. All images were corrected for photon attenuation, scatter, and radioactive decay and smoothed for uniform resolution [30]. Image analysis was done blind to clinical data. For each subject, summed PET images corresponding to 40 - 60 min of FDG data and 60 - 90 min of PiB data were coregistered to MRI using the Normalized Mutual Information (NMI) routine of Statistical Parametric Mapping (SPM8) [31]. Parametric standardized uptake value ratio (SUVR) images were generated by normalizing PiB uptake by cerebellar grey matter uptake and FDG by whole brain activity. MRIs were segmented into grey (GM), white matter (WM) and cerebrospinal fluid (CSF) and normalized to Montreal Neurological Institute (MNI) space by high-dimensional warping (DARTEL) using voxel-based morphometry, VBM8 [31] [32]. MRI-coregistered PET scans were spatially normalized using subject-specific transformation matrixes obtained from MRI, and smoothed with a 10 mm FWHM filter. MRIs were processed using VBM8. A custom template was created using MRI from all subjects by normalizing and segmenting the MRIs using the unified segmentation model with the MNI template and tissue probability maps (TPMs), and averaging the normalized subject TPMs [31]. Individual scans were then processed using the custom TPMs. Jacobian modulation was applied to restore absolute GM volumes (GMV) in the GM images, which were smoothed with an 8-mm FWHM kernel and normalized to total intracranial volumes.

### 2.5. Statistical Analysis

SPSS v.21 (SPPS Inc., 2013) and PLS Tool v1.0 implemented using MATLAB v7.2.0 (StatSoft Inc.) were used for analysis. The General Linear Model (GLM) and χ^2^ tests were used to compare clinical and demographical measures across LTA and MeDi groups (p < 0.05). Multivariate Partial Least Squares (PLS) regression analysis as implemented in PLS v1.0 was used for image analysis to compare LTA (LTA+ vs. LTA−), MeDi (MeDi+ vs. MeDi−) and LTA × MeDi combinations [33]-[35]. PLS regression is a multivariate extension of the multiple linear regression model that is used to construct predictive models when the number of variables (e.g., voxels) are far larger than the number of observations (e.g. subjects), and multi-collinear, as previously described [33]-[35]. Briefly, while univariate analysis is used to identify reliable signal changes at the level of individual image elements (*i.e.*, voxels), multivariate analysis focuses on the examination of distributed patterns by taking advantage of the spatial and/or temporal dependencies (*i.e.*, covariance) among image elements, thus enabling inferences about differences across space and/or time. The PLS procedure generates a latent variable (LV) brain map that includes all brain regions that are most correlated with the predicting variable (e.g., condition, or group), thus yielding spatial patterns of brain biomarkers that represent the optimal association between the images and the grouping factors [33]. The strength of the relationship is measured by the correlation between conditions and LV scores in the data. The final LV is a numeric score or value that corresponds to the expression of a voxel-pattern or, if only discrete variables are used, a mathematical combination of those discrete variables, for each subject [33].

For each modality (PiB, FDG, MRI), PLS analysis was performed to identify spatial patterns of brain biomarkers that represent the optimal association between brain images and grouping factors (LTA, MeDi, LTA × MeDi). LV scores were extracted from each significant pattern. Age, gender, education, ethnicity, BMI, APOE status, family history, presence of hypertension and HOMA scores were examined as covariates. The General linear model with post-hoc t-tests was used to compare LV scores across LTA × MeDi groups in post-hoc testing of interaction effects. All results were considered significant at p < 0.05. Multivariate analysis controls for the multiple chances to find group differences, and it does so without assuming independence of the dependent variables, yielding corrected *p* values. A gray segment mask derived from the MNI template was applied to the PiB and FDG scans during PLS analysis to focus the analysis on gray matter voxels. Split-half resampling was used to obtain unbiased measures of brain-LV reproducibility and prediction of the PLS model for independent data [35], whereby for each of the 2-group LTA and MeDi, and 4-group LTA × MeDi evaluations, five additional PLS results were generated using five different randomized combinations of 50% of the subjects from each group. These combinations included completely distinct sub-populations [35]. The resulting patterns were then compared for commonality across the six combinations of subjects, before and after resampling, to increase stability of results and provide indication of generalizability [35]. Anatomical location of brain regions showing significant effects was described using Talairach and Tournoux coordinates, after conversion from MNI space.

## 3. Results

### 3.1. Clinical Measures

Subjects’ characteristics are found in **Table 1**. None of the participants were diabetics, regular smokers, or met criteria for obesity as defined by a Body-Mass index (BMI) > 30 kg/m^2^. Thirty subjects (67%) were taking no medications. The remaining 15 subjects reported taking either or a combination of the following medications: high blood pressure medications (beta-, angiotensin receptor-, calcium channel-blockers, ACE inhibitors) and/or statins (16%), anti-depressants/SSRI (4%; note: all subjects washed off anti-depressants for at least 2 weeks before FDG-PET), prostate medications (4%), hormone replacement therapy (9%).

Of the 45 participants, 21 (47%) were more physically active (LTA+) and 24 (53%) were less physically active (LTA−). Eighteen (40%) showed higher adherence to the MeDi (MeDi+) and 27 (60%) showed lower adherence (MeDi−). The combination of physical activity and diet resulted in 4 sub-groups: physically active with higher MeDi adherence (LTA+/MeDi+; n = 7, 16%), physically active with lower MeDi adherence (LTA+/MeDi−; n = 14, 30%), less physically active with higher MeDi adherence (LTA−/MeDi+; n = 12, 27%) and less physically active with lower MeDi adherence (LTA−/MeDi−; n = 12, 27%).

Groups were comparable for clinical, demographical and neuropsychological measures (**Table 1**). There was a non-significant trend towards a higher frequency of women vs. men in LTA− vs. LTA+ (83% vs. 57%, p = 0.09), and in MeDi+ vs. MeDi− groups (83% vs. 63%, n = 0.14, n.s.). The MeDi− group included slightly more individuals with hypertension than the MeDi+ group (33% vs. 12%, p = 0.11).

### 3.2. PiB-PET

#### Physical activity (Figure 1 (A))

Significant differences in PiB retention were observed between LTA− and LTA+ groups (p < 0.001). The spatial pattern that provided the best separation between LTA groups included the precuneus, posterior cingulate, parietal, frontal, temporal and occipital cortex of both hemispheres (R^2^ = 0.33, p < 0.001**)**. This pattern was characterized by greater PiB retention, reflecting increased A*β* load, in LTA− as compared to LTA+ subjects. Age and HOMA scores were the only covariates that showed significant associations with the identified pattern (R^2^ = 0.09, p < 0.05). Including these variables in the model as covariates did not attenuate the strength of the association (R^2^ = 0.39, p < 0.001).

#### Diet (Figure 1 (B))

Significant differences in PiB retention were observed between MeDi− and MeDi+ groups (p < 0.001). The spatial pattern that provided the best separation between MeDi groups included frontal and temporal cortex, insula, and putamen of both hemispheres (R^2^ = 0.39, p < 0.001). This pattern was characterized by greater PiB retention in MeDi− as compared to MeDi+ subjects. Age was borderline associated with the identified pattern (R^2^ = 0.06, p ≤ 0.09). Correcting for age left results unchanged (R^2^ = 0.39, p < 0.001).

#### Physical activity × Diet (Figure 1 (C))

There were significant associations between increasing lifestyle risk and PiB retention (p < 0.001). The spatial pattern that provided the best separation between LTA × MeDi sub-groups included the precuneus, posterior cingulate, frontal and parieto-temporal cortex of both hemispheres, and left occipital gyrus, and was characterized by increasingly higher PiB retention as: LTA−/MeDi− > LTA−/MeDi+ > LTA+/MeDi− > LTA+/MeDi+ (R^2^ = 0.40, p < 0.001). On post-hoc analysis, the LTA−/MeDi− group had higher PiB LV scores than LTA+/MeDi+ (p < 0.001) and LTA+/MeDi− groups (p = 0.005); and the LTA−/MeDi+ group had higher PiB LV scores than the LTA+/MeDi+ group (p = 0.01) and marginally higher LV scores than the LTA+/MeDi− group (p = 0.07).Among covariates, age and HOMA scores showed trends towards associations with the identified pattern (R^2^ ≤ 0.08, p ≤ 0.10). Including these variables as covariates did not attenuate the strength of the association (R^2^ = 0.47, p < 0.001). Despite the significant correlations, the interaction of LTA × MeDi factors did not reach statistical significance, with and without correcting for covariates.

#### Resampling

Split-half resampling confirmed results obtained with the entire data set, with consistently higher PiB retention in AD-vulnerable regions for the higher-risk vs. lower-risk groups (p < 0.001). The greatest consistency across split half combinations was found for the LTA− vs. LTA+, and LTA−/MeDi− vs. LTA+/MeDi+ comparisons. Anatomical location and coordinates of significant clusters are found in **Table 2**.

### 3.3. FDG-PET

#### Physical activity

**Figure 2:**
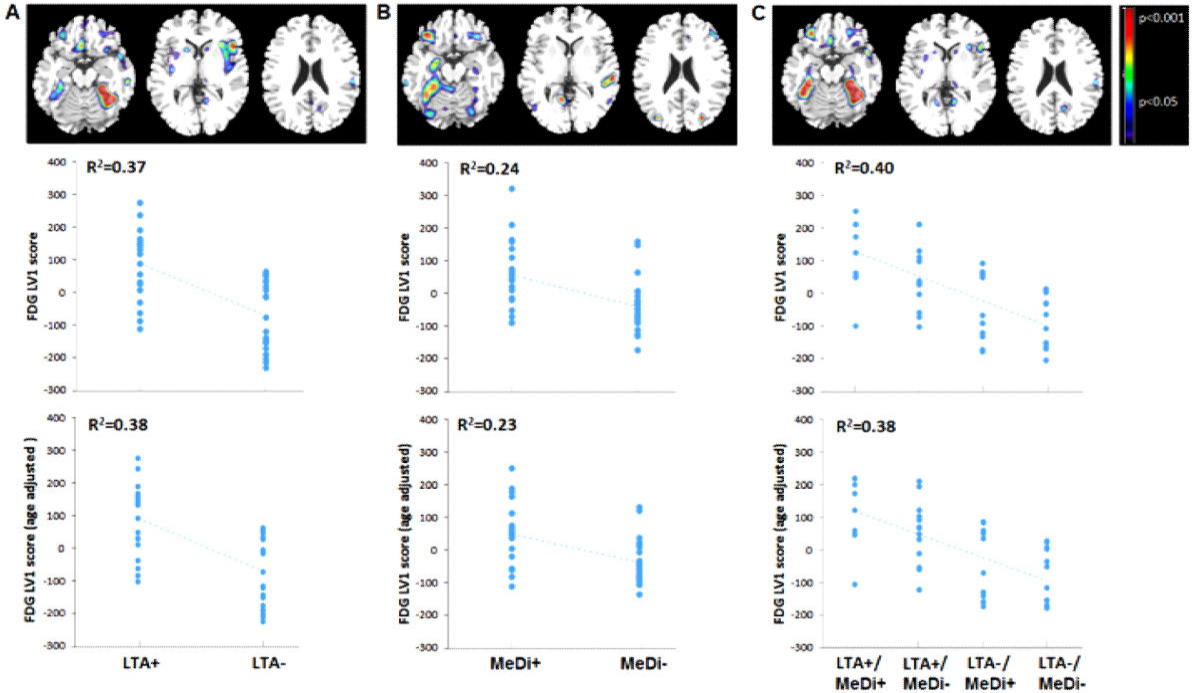
Spatial patterns of CMRglc as a function of physical activity and diet on FDG-PET. *Top row:* Partial Least Square regression maps (PLS maps; spatial biomarker patterns) that represent the optimal association between FDG-PET images and (A) leisure time physical activity (LTA), (B) Mediterranean Diet (MeDi), and (C) the combination of LTA and MeDi. P values are shown on a color-coded scale to the right. *Middle row:* LV scores extracted from each significant pattern show reduced FDG uptake, reflecting reduced CMRglc, in NL showing lower vs. higher engagement in leisure time physical activity (LTA− < LTA+); in NL showing lower vs. higher adherence to the MeDi (MeDi− < MeDi+) and as a function of LTA × MeDi (LTA−/MeDi− < LTA−/MeDi+ < LTA+/MeDi− < LTA+/MeDi+). R^2^ values are reported for each figure (all p’s < 0.001). *Bottom row:* LV scores are adjusted by age.

Significant CMRglc differences were observed between LTA− and LTA+ groups (p < 0.001). The spatial pattern that provided the best separation between LTA groups included lateral and medial temporal cortex (parahippocampal gyrus and uncus), fusiform gyrus and inferior frontal cortex of both hemispheres (R^2^ = 0.37, p < 0.001, **Figure 2(A)**). This pattern was characterized by reduced CMRglc in LTA− as compared to LTA+ subjects (**Figure 2(A)**). Age and HOMA scores showed borderline associations with the identified pattern (R^2^ ≤ 0.11, p ≤ 0.09). Including these variables as covariates in the model did not attenuate the strength of the association (covariates: age, R^2^ = 0.38, p < 0.001; HOMA scores, R^2^ = 0.40, p < 0.001).

#### Diet

Significant CMRglc differences were observed between MeDi− and MeDi+ groups (p < 0.001). The spatial pattern that provided the best separation between MeDi groups included broadly the same regions as with the LTA groups, but was primarily restricted to the lefthemisphere (R^2^ = 0.24, p < 0.001, **Figure 2(B)**). This pattern was characterized by reduced CMRglc in MeDi− as compared to MeDi+ subjects. Age was the only covariate associated with the identified pattern (R^2^ = 0.18, p = 0.003). Correcting for age left results unchanged (R^2^ = 0.23, p < 0.001, **Figure 2(B)**).

#### Physical activity × Diet

There were significant associations between increasing risk and reduced CMRglc (p < 0.001), as well as significant interaction effects of LTA × MeDi on CMRglc (p = 0.03). The spatial pattern that provided the best separation between LTA × MeDi subgroups included the same brain regions as above (e.g., lateral and medial temporal cortex, inferior frontal cortex, bilaterally) and was characterized by increasingly lower CMRglc, as: LTA−/MeDi− < LTA−/MeDi+ < LTA+/MeDi− < LTA+/MeDi+ (R^2^ = 0.40, p < 0.001, **Figure 2 (C)**). Interaction effects were driven by the LTA+/MeDi+ group showing higher FDG LV scores than all other groups (p < 0.05); and the LTA+/MeDi− group showing higher FDG LV scores than LTA−/MeDi− and LTA−/MeDi+ groups (p ≤ 0.04). Results remained significant correcting for age (p < 0.05, **Figure 2 (C)**). None of the other covariates showed significant associations with the identified pattern.

#### Resampling

Split-half resampling confirmed results obtained with the entire data set, with consistently lower CMRglc, especially in medial temporal cortex, for the higher-risk vs. lower-risk groups (p < 0.001 for main effects, p < 0.05 for interaction effects). The greatest consistency across split half combinations was observed for the LTA− vs. LTA+, and LTA−/MeDi− vs. LTA+/MeDi+ comparisons (p < 0.001).Anatomical location and coordinates of significant clusters are found in **Table 3**.

### 3.4. MRI

#### Physical activity

**Figure 3:**
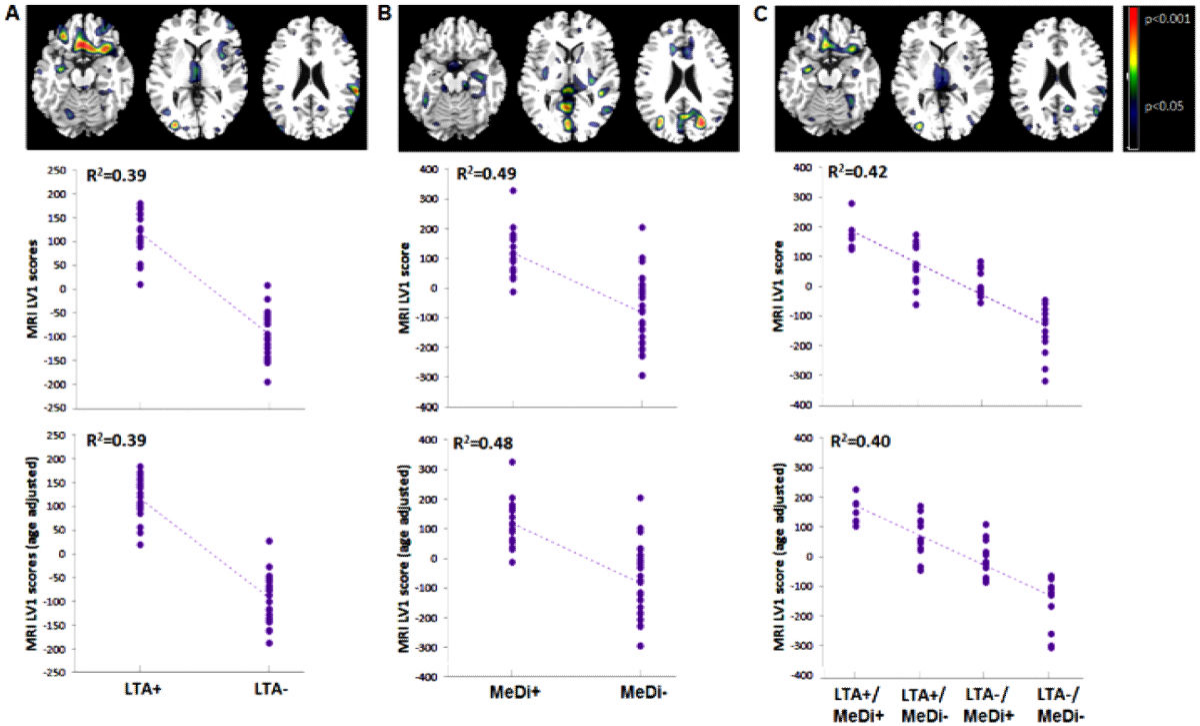
Spatial patterns of gray matter volumes as a function of physical activity and diet on MRI. *Top row:* Partial Least Square regression maps (PLS maps; spatial biomarker patterns) that represent the optimal association between GMV-MRI images and (A) leisure time physical activity (LTA), (B) Mediterranean Diet (MeDi), and (C) the combination of LTA and MeDi. P values are shown on a color-coded scale to the right. *Middle row:* LV scores extracted from each significant pattern show reduced GMV, reflecting increased atrophy, in NL showing lower vs. higher engagement in leisure time physical activity (LTA− < LTA+); in NL showing lower vs. higher adherence to the MeDi (MeDi− < MeDi+) and as a function of LTA × MeDi (LTA−/MeDi− < LTA−/MeDi+ < LTA+/MeDi− < LTA+/MeDi+). R^2^ values are reported for each figure (all p’s < 0.001). *Bottom row:* LV scores are adjusted by age.

Significant differences in GMV were observed between LTA− and LTA+ groups (p < 0.001). The spatial pattern that provided the best group separation included the superior and orbital frontal cortex, and cerebellum (R^2^ = 0.397, p < 0.001, **Figure 3(A)**). This pattern was characterized by reduced GMV, reflecting increased atrophy, in frontal cortex, and relative GMV increases in cerebellum, of LTA− as compared to LTA+ subjects. None of the covariates showed significant associations with the identified pattern.

#### Diet

Significant differences in GMV were observed between MeDi− and MeDi+ groups (p < 0.001). The spatial pattern that provided the best separation between MeDi groups included posterior cingulate cortex and precuneus, frontal and temporal cortex, and cerebellum (R^2^ = 0.49, p < 0.001, **Figure 3(B)**). This pattern was characterized by reduced cortical GMV, and relative cerebellar GMV increases, in MeDi− as compared to MeDi+ subjects. Gender was associated with the identified pattern (R^2^ = 0.11, p = 0.02), and age showed borderline effects (R^2^ = 0.05, p = 0.11). Including these variables as covariates in the model did not attenuate the strength of the association (covariates: age, R^2^ = 0.48, p < 0.001; gender, R^2^ = 0.40, p < 0.001).

#### Physical activity × Diet

There were significant associations between increasing risk and reduced GMV (p < 0.001). The spatial pattern that provided the best separation between LTA × MeDi subgroups included the superior, medial frontal and to a lesser extent, posterior cingulate cortex, and was characterized by increasingly lower GMV, as: LTA−/MeDi− < LTA−/MeDi+ < LTA+/MeDi− < LTA+/MeDi+ (R^2^ = 0.42, p < 0.001, **Figure 3 (C)**). On post-hoc analysis, the LTA−/MeDi− group had lower GMV LV scores than LTA+/MeDi+ and LTA+/MeDi− groups (p < 0.07). None of the covariates showed significant associations with the identified pattern. Despite the significant correlations, the interaction of LTA × MeDi factors did not reach statistical significance, with and without correcting for covariates.

#### Resampling

Split-half resampling confirmed the relative GMV decreases in cerebellum found in MeDi and LTA × MeDi comparisons for the higher-risk vs. lower-risk groups (p < 0.01), and lack of interaction effects overall. However, split half comparisons showed greater variability and lower concordance of other regional effects across combinations than with PiB and FDG data. It was noted that the clusters found to be significant in the group comparisons as a whole were less in extent than those observed with PiB or FDG. Anatomical location and coordinates of significant clusters are found in **Table 4**.

### 3.5. Clinical Measures and LV Scores

PiB LV scores from the *LTA pattern* were positively associated with HOMA scores and plasma triglycerides (r = 0.31 and 0.32, respectively, p < 0.05) and negatively, though weakly associated with HDL/LDL ratios (r = −0.26, p = 0.09). CMRglc LV scores from the *LTA pattern* were negatively associated with HOMA scores (r = −0.33), plasma triglycerides (r = −0.36) and HDL/LDL ratios (r = −0.36, p < 0.04). CMRglcLV scores from the *LTA* × *MeDi pattern* were also negatively associated with HDL/LDL ratios (r = −0.33, p < 0.03). GMV LV scores were not associated with any clinical measures. There were no associations between LV scores and neuropsychological measures for any biomarkers.

## 4. Discussion

This multi-modality brain imaging study provides evidence for favorable effects of LTA and MeDi on AD-biomarkers among NL adults. Less physically active subjects, and those showing lower MeDi adherence had increased AD-burden (*i.e.*, higher A*β* load, reduced CMRglc and GMV) as compared to their lower-risk counterparts. Moreover, NL subjects who were less physically active and showed lower MeDi adherence had the highest levels of AD-burden.

### Diet

A large body of evidence shows a favorable relation of a MeDi−type diet with slower cognitive decline, lower risk of AD, and reduced mortality in AD patients [4]-[6]. These effects were independent of physical activity [14] and were not mediated by vascular comorbidity [36]. Present findings confirm an association between higher MeDi adherence and larger GMV in key brain regions for AD, including medial temporal cortex [9], and further extend these observations to PiB and FDG-PET, by showing lower A*β* load and higher CMRglc in MeDi− compared to MeDi− subjects.

### Physical activity

Several biomarker studies reported associations between higher levels of aerobic exercise, as estimated with the Walk Run Jog questionnaire, brain volumes [15] [17], lower PiB binding and higher CSF A*β*42 levels [16] [18]. On the other hand, two studies that examined LTA failed to report significant effects on AD-biomarkers [37] [38], although higher LTA scores were associated with higher cognitive scores [37] and fewer white matter lesions in NL elderly [38]. Both studies reported associations between higher lifelong intellectual activity, rather than physical activity, and a better AD biomarker profile, including lower PiB retention, higher FDG uptake and MRI-based hippocampal volumes [37] [38]. Lack of LTA effects may be due to methodological considerations. These negative studies used regions of interest instead of voxel based analysis (VBA), as performed in the present study, which has been shown to be more sensitive to detect early biomarker abnormalities [39]. Moreover, previous papers focused on an older population than in our study (mean age 79 [37] and 75 y [38], respectively, vs. 54 y). Present results show that young to late-middle aged NL adults who met the exercise guidelines set by the American Heart Association had significantly lower PiB retention, higher CMRglc and GMV in AD-regions, as well as a more favorable HDL/LDL ratios, lower insulin resistance and plasma triglycerides, as compared to more sedentary individuals. Future studies are needed to examine the combined effects of diet, physical activity and intellectual activity on AD biomarkers in NL adults.

### Diet and Physical activity

To our knowledge, there are no previous reports of the combined effects of physical activity and diet on AD biomarkers in NL adults. Clinical studies that examined this combination showed that physical activity and diet were independently associated with reduced risk for AD, while their combination did not result in further risk reductions relative to each factor alone [14]. In the present study, the combination of these lifestyle factors resulted in increasing levels of AD-burden, in that LTA+/MeDi+ subjects had the lowest levels, LTA−/MeDi+ and LTA+/MeDi− had intermediate levels, and LTA−/MeDi− subjects had the highest levels of AD-burden, with all three modalities. The interaction reached statistical significance for FDG, but not for PiB or MRI measures, although trends were noted. These data suggest that the primary effect of MeDi and LTA may be one of increased bio-energetics, although this remains to be confirmed. While brain imaging measures of AD pathology and associated neuronal injury are increasingly used for clinical purposes, other techniques are needed to specifically assess the molecular mechanisms behind the observed increases in PiB signal or reductions in FDG uptake, which are measured at the tissue, rather than at the cellular level [40]. At the molecular level, other studies have shown that aerobic activity might be neuroprotective through the regulation of brain-derived neurotrophic factor (BDNF) secretion [41], which is necessary for long-term potentiation and memory formation, and for the growth and survival of new neurons [42] [43]. Brain bioenergetics and age-associated energy crisis are increasingly thought to play an important role in late-onset AD [44]. Therefore, other markers of cellular respiration and metabolism, such as measurements of membrane stability, energy metabolism and activity of antioxidant enzymes from peripheral blood cells (*i.e.*, erythrocytes) [45] [46] may shed additional light on the complex mechanisms involved in lifestyle-based modulation of AD pathology during the normal stages of cognition.

Overall, present multi-modality brain imaging findings indicate that increased LTA and higher MeDi adherence may modulate AD risk through its effects on A*β* levels and associated neuronal injury in NL adults. These data further support novel treatment strategies aimed at delaying cognitive decline and modifying AD progression through simultaneous implementation of pharmacological and non-pharmacological interventions [47] [48]. Candidate non-pharmacological treatments include, but are not limited to, increased sensory input through physical and mental activities, which is directed at improving cerebral blood flow, and nutritional interventions such as diet modification, vitamin supplementation and nutraceuticals [48].

### Limitations

Given the relatively small sample, other studies with larger samples are needed to replicate these preliminary findings. Second, the PLS procedures do not enable direct comparison of the strength of the associations across biomarkers, and as such, we cannot conclude that lifestyle affects one modality more than another. It is possible that one lifestyle factor may have greater risk-lowering effects than the other, which could not be established in this study. Third, a causal or temporal relationship between lifestyle factors and AD biomarkers remains to be established. The purpose of this cross-sectional study was to provide preliminary evidence of physical activity and MeDi effects on multiple AD biomarkers in NL adults. Future longitudinal studies with larger samples are warranted to assess the causal relationship between lifestyle and AD-biomarkers, and whether this relationship varies with age and disease, thereby providing much needed data for randomized, clinical trials of lifestyle interventions. Moreover, this cross-sectional study does not provide evidence for differential clinical outcomes as a function of the participants’ diet and exercise status. The goal of this paper was to detect differential risk of AD as a function of lifestyle factors, as obtained from the use of biomarkers, in cognitively normal individuals who, by our inclusion criteria, did not present with cognitive deficits. Present findings set the stage for larger, longitudinal studies that will assess lifestyle-related changes in biomarkers, as well as in clinical or cognitive outcomes.

Approximately 35% of our NL participants were APOE *ε*4 carriers. As such, we did not have enough subjects to reliably test for interactions between lifestyle factors and APOE status. As previous studies have shown stronger effects of lifestyle factors on AD-biomarkers in APOE *ε*4 non-carriers [9] [16] [18], it is possible that the combination of MeDi and physical activity may also be stronger for the non-carriers.

Most, if not all participants reported stability of their dietary patterns and LTA over the past 2 - 5 years. Examination of our records showed that approximately 90% of the surveyed participants have been living the life-style reported in the surveys for 5 years or more, with a very conscientious focus on their diet and physical activity choices. Approximately 8% of those surveyed reported their nutritional intake to be a lifestyle span of about 2 - 5 years. Only 1 participant in the MeDi− group reported their nutritional behavior starting within the last 1 - 2 years. Overall, our MeDi+ cohort included people for whom the MeDi was their normal dietary pattern, and most of the MeDi+ participants reported following the MeDi since childhood. Previous longitudinal studies of the MeDi with repeated dietary assessments over up to 13 years, demonstrated that adherence to the MeDi is remarkably stable over time, especially in healthy individuals [4] [8]. However, while we consider it more likely that the lifestyle patterns reported reflects our population’s longstanding habits, because of the synchronous timing of dietary and brain imaging assessments and the cross-sectional nature of our study, we cannot exclude that these may be more recent lifestyle choices. Future studies are needed to test whether AD biomarkers change mostly after long-term exposure to healthy lifestyle patterns, or whether short-term exposure is sufficient.

Finally, we caution that the NL population selected in our study represents a group with a high a priori risk of preclinical AD-changes, and results were made with small numbers of carefully screened subjects under controlled clinical conditions. Replication of these preliminary research findings in community-based populations with more diversified socio-economic status is warranted and clinical application is not justified.

## 5. Conclusion

NL adults who engage in moderate physical activity and follow a MeDi− style diet had the lowest levels of brain AD-burden. In contrast, sedentary individuals with lower MeDi adherence had the highest levels of AD-burden, years in advance of possible clinical symptoms. Evidence for an association between lifestyle and AD risk during the normal stages of cognition provides support for further exploration of lifestyle modifications for the prevention of AD.

## Figures and Tables

**Figure 1:**
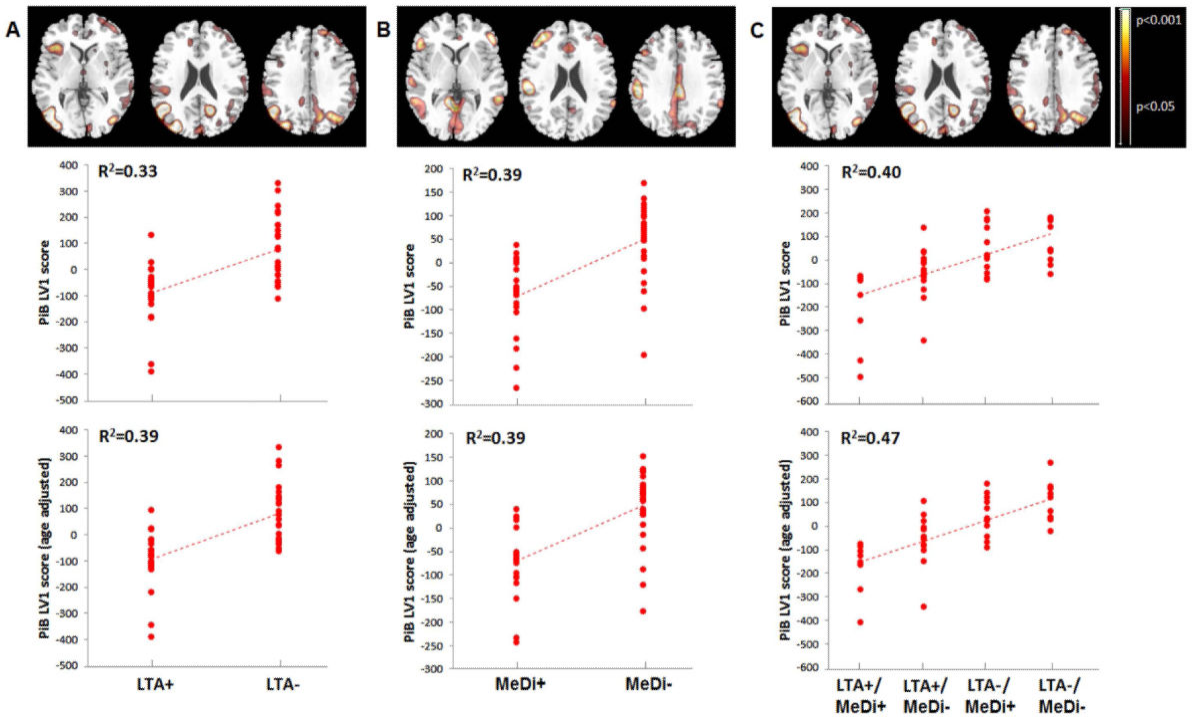
Spatial patterns of brain A*β* load as a function of physical activity and diet on PiB-PET. *Top row:* Partial Least Square regression maps (PLS maps; spatial biomarker patterns) that represent the optimal association between PiB-PET images and (A) leisure time physical activity (LTA), (B) Mediterranean Diet (MeDi), and (C) the combination of LTA and MeDi. P values are shown on a color-coded scale to the right. *Middle row:* LV scores extracted from each significant pattern show increased PiB retention, reflecting higher A*β* load, in NL showing lower vs. higher engagement in leisure time physical activity (LTA− > LTA+); in NL showing lower vs. higher adherence to the MeDi (MeDi− > MeDi+) and as a function of LTA × MeDi (LTA−/MeDi− > LTA−/MeDi+ > LTA+/MeDi− > LTA+/MeDi+). R^2^ values are reported for each figure (all p’s < 0.001). *Bottom row:* LV scores are adjusted by age.

**Table 1:** Demographic and clinical characteristics.

	LTA		MeDi	

	LTA+	LTA−	MeDi+	MeDi−
N	21	24	18	27
Age, y, mean (SD)	54 (11)	54 (11)	54 (11)	54 (11)
Female gender, %	57%	83%	83%	63%
Education, y, mean (SD)	16 (2)	16 (2)	16 (2)	16 (2)
Family history of LOAD, % positive	62%	79%	72%	70%
APOE ε4 status*, % positive	33%	50%	53%	36%
Ethnicity, % White/Non-Hispanic	71%	92%	78%	85%
Hypertension, % yes	26%	23%	12%	33%
**Anthropometrics and Labs, mean (SD)**				
Body mass index [unitless]	26 (4)	25 (4)	24 (4)	26 (5)
Hip to waist ratio [unitless]	1.4 (0.1)	1.5 (0.4)	1.0 (0.3)	1.2 (0.2)
Blood pressure (mm/Hg) Systolic Diastolic	119 (15) 68 (12)	120 (15) 74 (7)	122 (12) 75 (8)	119 (16) 70 (15)
HOMA score (mg/dl)	1.1 (0.6)	1.8 (2.5)	1.8 (2.1)	1.3 (2.1)
Cholesterol (mg/dl)	207 (43)	195 (36)	207 (44)	197 (35)
Triglycerides (mg/dl)	86 (40)	95 (37)	98 (44)	85 (33)
HDL/LDL [unitless]	0.59 (0.22)	0.51 (0.17)	0.54 (0.16)	0.56 (0.22)
Homocysteine (micromol/l)	10.6 (2.3)	10.0 (2.8)	10.8 (3.1)	9.8 (2.1)
**Neuropsychological Tests, mean (SD)**				
Mini mental state exam	29 (1)	29 (1)	29 (1)	29 (1)
Digit symbol substitution	65 (11)	61 (9)	63 (10)	62 (11)
Paired associates delayed recall	7 (3)	7 (3)	7 (2)	6 (3)
Paragraph delayed recall	10 (3)	10 (2)	10 (3)	10 (3)
Designs	8 (2)	8 (2)	8 (2)	8 (2)
Object naming	55 (12)	53 (13)	55 (12)	53 (13)
WAIS-vocabulary	66 (9)	62 (15)	66 (10)	62 (18)

Abbreviations: MeDi = adherence to a Mediterranean-type diet (higher vs. lower, MeDi+ vs. MeDi−); LTA = engagement in leisure time activity (higher vs. lower, LTA+ vs. LTA−).

**Table 2:** Brain regions included in PLS-derived lifestyle patterns using PiB-PET.

Cluster Extent	Coordinates (x, y, z)*	Anatomical Area, Brodmann Area (BA)
**Physical Activity Pattern (LTA− vs.** LTA+)		
2778	−41, −73, −4	Left Cerebrum, Inferior Occipital Gyrus, BA 19
	−46, −69, 4	Left Cerebrum, Middle Occipital Gyrus, BA 37
363	37, −78, −11	Right Cerebrum, Occipital Lobe, Fusiform Gyrus, BA 19
553	43, 33, 15	Right cerebrum, Frontal lobe, Middle Frontal Gyrus, BA 46
765	57, −16, 11	Right cerebrum, Temporal lobe, Transverse Temporal Gyrus, BA 42
700	14, −57, 12	Right cerebrum, Limbic lobe, Posterior Cingulate, BA 30
433	−46, −61, 45	Left cerebrum, Parietal lobe, Inferior Parietal Lobule, BA 40
258	−25, −69, 38	Left cerebrum, Parietal lobe, Precuneus, BA 7
**MeDi Pattern (MeDi− vs. MeDi+)**		
2014	−29, −3, −3	Left Cerebrum, Sub-Lobar, Putamen
	−41, −5, 1	Left Cerebrum, Insula
633	4, 51, −4	Right Cerebrum, Frontal lobe, Medial Frontal Gyrus, BA 10
214	46, −65, 7	Right Cerebrum, Temporal Lobe, Middle Temporal Gyrus, BA 37
308	28, −3, −3	Right Cerebrum, Sub-Lobar, Putamen
**Physical Activity × MeDi Pattern (LTA−/MeDi− vs. LTA-/MeDi+ vs. LTA+/MeDi− vs. LTA+/MeDi+)**		
3265	−40, −73, 6	Left Cerebrum, Occipital Lobe, Middle Occipital Gyrus, BA 19
	−46, −65, 7	Left Cerebrum, Temporal Lobe, Middle Temporal Gyrus, BA 37
213	−39, −57, 17	Left Cerebrum, Temporal Lobe, Middle Temporal Gyrus, BA 22
226	36, −63, 27	Right Cerebrum, Temporal Lobe, Middle Temporal Gyrus, BA 39
298	13, −58, 24	Right Cerebrum, Parietal Lobe, Precuneus, BA 31
232	13, −61, 17	Right Cerebrum, Limbic Lobe, Posterior Cingulate Gyrus, BA 31

**Table 3:** Brain regions included in PLS-derived lifestyle patterns using FDG-PET.

Cluster Extent	Coordinates (x, y, z)*	Anatomical Area, Brodmann Area (BA)
**Physical Activity Pattern (LTA− vs. LTA+)**		
2307	21, −5, −33	Right Cerebrum, Limbic Lobe, Uncus, BA 36
	32, −24, −26	Right Cerebrum, Limbic Lobe, Parahippocampal Gyrus, BA 36
	36, −49, −16	Right Cerebrum, Temporal Lobe, Fusiform Gyrus, BA 37
	41, 12, −29	Right Cerebrum, Temporal Lobe, Superior Temporal Gyrus, BA 38
	44, 8, −28	Right Cerebrum, Temporal Lobe, Middle Temporal Gyrus, BA 21
210	−29, −24, −24	Left Cerebrum, Limbic Lobe, Parahippocampal Gyrus, BA 36
197	44, 18, 2	Right Cerebrum, Inferior Frontal Gyrus, BA 47
**MeDi Pattern (MeDi− vs. MeDi+)**		
908	−25, −36, −12	Left Cerebrum, Limbic Lobe, Parahippocampal Gyrus, BA 36
**226**	−16, −9, −20	Left Cerebrum, Limbic Lobe, Parahippocampal Gyrus, BA 34
**228**	20, −71, −10	Right Cerebrum, Occipital Lobe, Lingual Gyrus, BA 18
263	55, −28, −1	Right Cerebrum, Temporal Lobe, Middle Temporal Gyrus, BA 21
**Physical Activity × MeDi Pattern (LTA−/MeDi− vs. LTA−/MeDi+ vs. LTA+/MeDi− vs. LTA+/MeDi+)**		
1174	34, −47, −16	Right Cerebrum, Temporal Lobe, Fusiform Gyrus
948	−25, −38, −14	Left Cerebrum, Temporal Lobe, Parahippocampal Gyrus
	−29, −38, −18	Left Cerebrum, Temporal Lobe, Fusiform Gyrus, BA 20
931	25, −3, −37	Right Cerebrum, Limbic Lobe, Uncus, BA 20
	32, 12, −36	Right Cerebrum, Temporal Lobe, Superior Temporal Gyrus, BA 38

See legend to **Table 2.**

**Table 4:** Brain regions included in PLS-derived lifestyle patterns using MRI.

Cluster Extent	Coordinates (x, y, z)*	Anatomical Area, Brodmann Area (BA)
**Physical Activity Pattern (LTA− vs. LTA+)**		
350	10, 68, 14	Right Cerebrum, Frontal Lobe, Superior Frontal Gyrus, BA 10
305	−28, −66, −36	Left Cerebellum, Posterior Lobe, Inferior Semilunar Lobule
**MeDi Pattern (MeDi− vs. MeDi+)**		
578	46, −2, −46	Right Cerebrum, Temporal Lobe, Inferior Temporal Gyrus, BA 20
261	62, 8, 18	Right Cerebrum, Frontal Lobe, Inferior Frontal Gyrus, BA 44
1199	2, −52, −32	Right Cerebellum, Posterior Lobe, Cerebellar Tonsil
207	−8, −54, −4	Left Cerebellum, Anterior Lobe, Culmen
**Physical Activity × MeDi Pattern (LTA−/MeDi− vs. LTA−/MeDi+ vs. LTA+/MeDi− vs. LTA+/MeDi+)**		
788	−56, −10, 16	Left Cerebrum, Frontal Lobe, Precentral Gyrus, BA 13
443	−8, 64, −6	Left Cerebrum, Frontal Lobe, Superior Frontal Gyrus, BA 10
264	−6, 45, 10	Left Cerebrum, Frontal Lobe, Medial Frontal Gyrus, BA 10
1397	28, −68, −38	Right Cerebellum, Posterior Lobe, Inferior Semilunar Lobule

See legend to **Table 2.**

## References

[1] Barnes DE, Yaffe K (2011). The Projected Effect of Risk Factor Reduction on Alzheimer’s Disease Prevalence. The Lancet Neurology.

[2] Sperling RA, Karlawish J, Johnson KA (2013). Preclinical Alzheimer Disease—The Challenges Ahead. Nature Reviews Neurology.

[3] Jack CR, Knopman DS, Jagust WJ (2010). Hypothetical Model of Dynamic Biomarkers of the Alzheimer’s Pathological Cascade. The Lancet Neurology.

[4] Gu Y, Scarmeas N (2011). Dietary Patterns in Alzheimer’s Disease and Cognitive Aging. Current Alzheimer Research.

[5] Scarmeas N, Stern Y, Mayeux R, Manly JJ, Schupf N, Luchsinger JA (2009). Mediterranean Diet and Mild Cognitive Impairment. Archives of Neurology.

[6] Scarmeas N, Stern Y, Tang MX, Mayeux R, Luchsinger JA (2006). Mediterranean Diet and Risk for Alzheimer’s Disease. Annals of Neurology.

[7] Trichopoulou A, Costacou T, Bamia C, Trichopoulos D (2003). Adherence to a Mediterranean Diet and Survival in a Greek Population. New England Journal of Medicine.

[8] Feart C, Samieri C, Rondeau V (2009). Adherence to a Mediterranean Diet, Cognitive Decline, and Risk of Dementia. JAMA.

[9] Mosconi L, Murray J, Tsui WH (2014). Mediterranean Diet and Magnetic Resonance Imaging-Assessed Brain Atrophy in Cognitively Normal Individuals at Risk for Alzheimer’s Disease. Journal of Prevention of Alzheimer’s Disease.

[10] Scarmeas N, Luchsinger JA, Stern Y (2011). Mediterranean Diet and Magnetic Resonance Imaging-Assessed Cerebrovascular Disease. Annals of Neurology.

[11] Gardener H, Scarmeas N, Gu Y, Boden-Albala B, Elkind MSV, Sacco RL (2012). Mediterranean Diet and White Matter Hyperintensity Volume in the Northern Manhattan Study. Archives of Neurology.

[12] Hillman CH, Erickson KI, Kramer AF (2008). Be Smart, Exercise Your Heart: Exercise Effects on Brain and Cognition. Nature Reviews Neuroscience.

[13] Hayes SM, Hayes JP, Cadden M, Verfaellie M (2013). A Review of Cardiorespiratory Fitness-Related Neuroplasticity in the Aging Brain. Frontiers in Aging Neuroscience.

[14] Scarmeas N, Luchsinger JA, Schupf N, Brickman AM, Cosentino S, Tang MX, Stern Y (2009). Physical Activity, Diet, and Risk of Alzheimer Disease. JAMA.

[15] Burns JM, Cronk BB, Anderson HS, Donnelly JE, Thomas GP, Harsha A (2008). Cardiorespiratory Fitness and Brain Atrophy in Early Alzheimer Disease. Neurology.

[16] Head D, Bugg JM, Goate AM, Fagan AM, Mintun MA, Benzinger T (2012). Exercise Engagement as a Moderator of the Effects of *APOE* Genotype on Amyloid Deposition. Archives of Neurology.

[17] Honea RA, Thomas GP, Harsha A, Anderson HS, Donnelly JE, Brooks WM (2009). Cardiorespiratory Fitness and Preserved Medial Temporal Lobe Volume in Alzheimer Disease. Alzheimer Disease & Associated Disorders.

[18] Liang KY, Mintun MA, Fagan AM, Goate AM, Bugg JM, Holtzman DM (2010). Exercise and Alzheimer’s Disease Biomarkers in Cognitively Normal Older Adults. Annals of Neurology.

[19] De Santi S, Pirraglia E, Barr W, Babb J, Williams S, Rogers K (2008). Robust and Conventional Neuropsychological Norms: Diagnosis and Prediction of Age-Related Cognitive Decline. Neuropsychology.

[20] Lann D, LeRoith D (2007). Insulin Resistance as the Underlying Cause for the Metabolic Syndrome. Medical Clinics of North America.

[21] Glodzik L, Mosconi L, Tsui W, de Santi S, Zinkowski R, Pirraglia E (2012). Alzheimer’s Disease Markers, Hypertension, and Gray Matter Damage in Normal Elderly. Neurobiology of Aging.

[22] Mosconi L, Brys M, Switalski R, Mistur R, Glodzik L, Pirraglia E (2007). Maternal Family History of Alzheimer’s Disease Predisposes to Reduced Brain Glucose Metabolism. Proceedings of the National Academy of Sciences of the United States of America.

[23] Hixson JE, Powers PK (1991). Restriction Isotyping of Human Apolipoprotein A-IV: Rapid Typing of Known Isoforms and Detection of a New Isoform That Deletes a Conserved Repeat. Journal of Lipid Research.

[24] Taylor HL, Jacobs DR, Schucker B, Knudsen J, Leon AS, Debacker G (1978). A Questionnaire for the Assessment of Leisure Time Physical Activities. Journal of Chronic Diseases.

[25] Thompson PD, Buchner D, Piña IL, Balady GJ, Williams MA, Marcus BH (2003). Exercise and Physical Activity in the Prevention and Treatment of Atherosclerotic Cardiovascular Disease: A Statement from the Council on Clinical Cardiology (Subcommittee on Exercise, Rehabilitation, and Prevention) and the Council on Nutrition, Physical Activity, and Metabolism (Subcommittee on Physical Activity). Circulation.

[26] Strath SJ, Kaminsky LA, Ainsworth BE, Ekelund U, Freedson PS, Gary RA (2013). Guide to the Assessment of Physical Activity: Clinical and Research Applications: A Scientific Statement from the American Heart Association. Circulation.

[27] Willett WC, Sampson L, Stampfer MJ, Rosner B, Bain C, Witschi J (1985). Reproducibility and Validity of a Semiquantitative Food Frequency Questionnaire. American Journal of Epidemiology.

[28] Mosconi L, Murray J, Tsui W, Spector N, Goldowsky A, Williams S (2014). Brain Imaging of Cognitively Normal Individuals with 2 Parents Affected by Late-Onset AD. Neurology.

[29] Mosconi L, Andrews RD, Matthews DC, Alzheimer’s Disease Neuroimaging Initiative (2013). Comparing Brain Amyloid Deposition, Glucose Metabolism, and Atrophy in Mild Cognitive Impairment with and without a Family History of Dementia. Journal of Alzheimer’s Disease.

[30] Joshi A, Koeppe RA, Fessler JA (2009). Reducing between Scanner Differences in Multi-Center PET Studies. Neuroimage.

[31] Ashburner J, Friston KJ (2000). Voxel-Based Morphometry—The Methods. Neuroimage.

[32] Ashburner J (2007). A Fast Diffeomorphic Image Registration Algorithm. Neuroimage.

[33] Krishnan A, Williams LJ, McIntosh AR, Abdi H (2011). Partial Least Squares (PLS) Methods for Neuroimaging: A Tutorial and Review. Neuroimage.

[34] McIntosh AR, Lobaugh NJ (2004). Partial Least Squares Analysis of Neuroimaging Data: Applications and Advances. Neuroimage.

[35] Churchill N, Spring R, Abdi H, Kovacevic N, McIntosh AR, Strother S, Abdi H, Chin W, Vinzi E, Russolillo G, Trinchera L (2013). The Stability of Behavioral PLS Results in Ill-Posed Neuroimaging Problems. New Perspectives in Partial Least Squares and Related Methods.

[36] Scarmeas N, Stern Y, Mayeux R, Luchsinger JA (2006). Mediterranean Diet, Alzheimer Disease, and Vascular Mediation. Archives of Neurology.

[37] Vemuri P, Lesnick TG, Przybelski SA, Knopman DS, Roberts RO, Lowe VJ (2012). Effect of Life-style Activities on Alzheimer Disease Biomarkers and Cognition. Annals of Neurology.

[38] Wirth M, Haase CM, Villeneuve S, Vogel J, Jagust WJ (2014). Neuroprotective Pathways: Lifestyle Activity, Brain Pathology, and Cognition in Cognitively Normal Older Adults. Neurobiology of Aging.

[39] Mosconi L, Tsui WH, De Santi S, Li J, Rusinek H, Convit A (2005). Reduced Hippocampal Metabolism in MCI and AD: Automated FDG-PET Image Analysis. Neurology.

[40] Mosconi L (2013). Glucose Metabolism in Normal Aging and Alzheimer’s Disease: Methodological and Physiological Considerations for PET Studies. Clinical and Translational Imaging.

[41] Adlard PA, Perreau VM, Pop V, Cotman CW (2005). Voluntary Exercise Decreases Amyloid Load in a Transgenic Model of Alzheimer’s Disease. The Journal of Neuroscience.

[42] Cotman CW, Berchtold NC, Christie LA (2007). Exercise Builds Brain Health: Key Roles of Growth Factor Cascades and Inflammation. Trends in Neurosciences.

[43] Erickson KI, Voss MW, Prakash RS, Basak C, Szabo A, Chaddock L (2011). Exercise Training Increases Size of Hippocampus and Improves Memory. Proceedings of the National Academy of Sciences of the United States of America.

[44] Swerdlow RH (2012). Mitochondria and Cell Bioenergetics: Increasingly Recognized Components and a Possible Etiologic Cause of Alzheimer’s Disease. Antioxidants & Redox Signaling.

[45] Tikhonova LA, Kaminsky YG, Reddy VP, Li Y, Solomadin IN, Kosenko EA (2014). Impact of Amyloid β_25-35_ on Membrane Stability, Energy Metabolism, and Antioxidant Enzymes in Erythrocytes. American Journal of Alzheimer’s Disease and Other Dementias.

[46] Kaminsky YG, Reddy VP, Ashraf GM, Ahmad A, Benberin VV, Kosenko EA (2013). Age-Related Defects in Erythrocyte 2,3-Diphosphoglycerate Metabolism in Dementia. Aging and Disease.

[47] Bragin V, Chemodanova M, Dzhafarova N, Bragin I, Czerniawski JL, Aliev G (2005). Integrated Treatment Approach Improves Cognitive Function in Demented and Clinically Depressed Patients. American Journal of Alzheimer’s Disease and Other Dementias.

[48] Aliev G, Ashraf GM, Kaminsky YG, Sheikh IA, Sudakov SK, Yakhno NN (2013). Implication of the Nutritional and Nonnutritional Factors in the Context of Preservation of Cognitive Performance in Patients with Dementia/Depression and Alzheimer Disease. American Journal of Alzheimer’s Disease and Other Dementias.

